# A partially automated method for DNA extraction from marmoset hair follicles to avoid blood chimerism

**DOI:** 10.3389/fgene.2025.1608504

**Published:** 2025-09-26

**Authors:** Alexandra M. Stendahl, Qiangge Zhang, Ana C. Lima, Curtis Mello, James Nemesh, Sam Peterson, Jenna Castro, Fritzie T. Celino-Brady, Karina Ray, Xian Gao, Yuanyuan Hou, Chenjie Shen, Katinka A. Vigh-Conrad, Fenna Krienen, Guoping Feng, Steven A. McCarroll, Donald F. Conrad, Ricardo C. H. del Rosario

**Affiliations:** ^1^ Department of Genetics, Oregon National Primate Research Center, Beaverton, OR, United States; ^2^ Department of Brain and Cognitive Sciences, McGovern Institute for Brain Research, Massachusetts Institute of Technology (MIT), Cambridge, MA, United States; ^3^ Stanley Center for Psychiatric Research, Broad Institute of MIT and Harvard, Cambridge, MA, United States; ^4^ Harvard Medical School, Department of Genetics, Boston, MA, United States

**Keywords:** hair follicles, DNA extraction, maxwell RSC, chimerism, marmoset

## Abstract

Marmosets are valuable non-human primate models, however their unique reproduction results in high levels of blood chimerism, making blood unreliable for DNA sequencing. Hair follicles have lower levels of chimerism however DNA extraction from hair follicles is challenging due to the limited tissue. We developed a non-invasive, partially automated protocol for hair follicle collection and DNA extraction scalable to hundreds of samples. This method uses a proteinase K cell lysis solution in conjunction with Promega’s Maxwell RSC’s paramagnetic silica-based particles to purify DNA. We applied this protocol to samples collected from over 300 animals and from two different species. We were able to generate high quality libraries for whole genome sequencing (WGS) from approximately 150 hair follicles. Libraries built from >0.15 µg DNA had an average duplication rate of 0.19, analogous to libraries built from blood. Sequenced DNA had average chimerism rates of 2.3%. DNA extraction from hair follicles offers a reliable method for whole genome sequencing with minimal chimerism. The partial automation improves efficiency by reducing lab time and extraction variability. The protocol is applicable to a range of projects requiring low-input DNA sources or automated, high-throughput sample processing. Peer review data availability: Data on DNA yields and resulting whole genome sequencing libraries are provided as Supplementary Tables. The raw whole genome sequencing data produced from these libraries are archived online at the NIH Sequence Read Archive.

## 1 Introduction

The common marmoset (*Callithrix jacchus*) is an emerging, valuable non-human primate model for translational neuroscience because of its high fertility, short lifespan, and humanoid brain anatomy. Recent advances in its genome assembly and genetic engineering allow many kinds of genetic approaches to be extended to marmosets.

**FIGURE 1:**
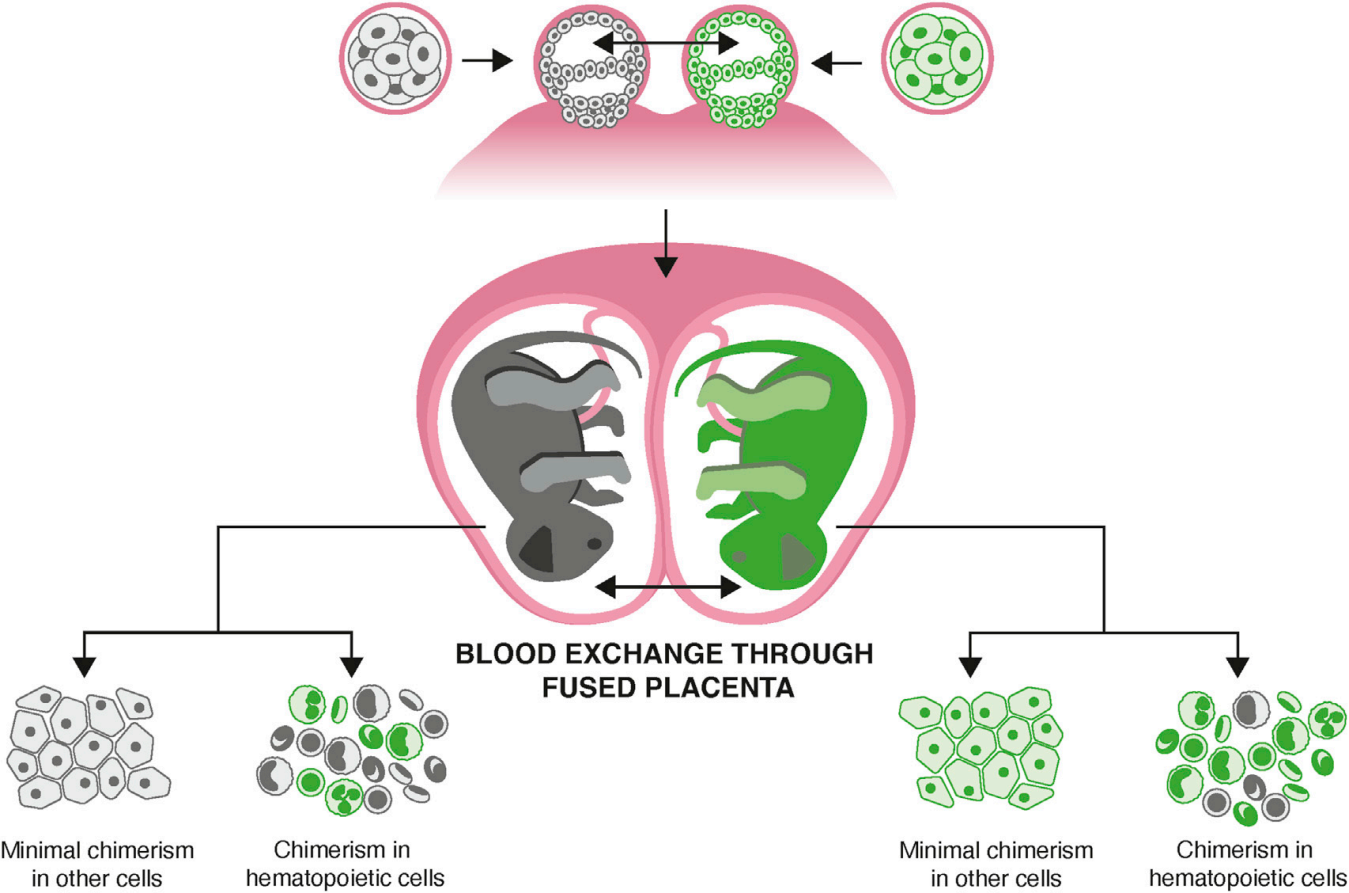
Hematopoietic stem cell mixing *in utero* causing blood chimerism. Marmosets have a high frequency of fraternal twinning. Vascular anastomoses of the placenta lead to exchange of hematopoietic stem cells between animals. These engraft in bone marrow, leading to life-long chimerism in every animal, which affects all hematopoietic lineages, including those resident in solid tissues.

However, the marmoset’s unique reproduction results in blood chimerism, posing significant challenges for genetic analysis (Benirschke et al., 1962; Gengozian et al., 1964; Kim et al., 2024). Marmosets reproduce by multiple gestation, with high rates of fraternal twinning routinely leading to litters of 2-3 animals in captivity. Placental anastomoses form during fetal development, leading to the exchange of hematopoietic stem cells, which invariably engraft in the bone marrow of each sibling (Figure 1).

Blood chimerism is approximately 50% in affected individuals, complicating the use of blood-derived DNA for host genome sequencing. Single-cell RNA-sequencing indicates that chimerism is limited to the hematopoietic lineage; however, many solid tissues contain some varying proportion of hematopoietic cells (del Rosario et al., 2024). Tissues such as skin and hair follicles show lower levels of chimerism, with 9%–24% in buccal tissue and closer to 1% in hair follicles. It is possible to obtain non-chimeric DNA by culturing fibroblasts from skin biopsies (Harris et al., 2023). However, fibroblast culture requires many additional steps, involves surgical sampling of tissue, and is expensive. Hair follicles in particular are an attractive source of DNA, as they can be sampled without surgery or needles, but they pose challenges for DNA extraction due to the limited amount of cells present. Currently, no routine, automated method exists that extracts sufficient DNA from hair follicles for whole genome sequencing (WGS).

As part of our ongoing work with marmosets colonies, we developed an easy, non-invasive, partially automated, protocol for the collection of hair follicles and extraction of DNA with minimal chimerism. Researchers can collect hair follicles during routine veterinary exams, which can be sent on ice for extraction and sequencing.

Hair follicles have previously been used for DNA extraction in animal models, especially in marmosets, but existing methods are not easily scalable for large sample sizes. Furthermore, most protocols were only used to genotype specific SNPs rather than generate a library suitable for whole genome sequencing (Cole et al., 2024). Used 50–100 hair follicles and the QIAmp DNA Mini Kit to identify specific SNPs (Améndola-Pimenta et al., 2009). Used hair follicles from wild primates and a proteinase-K lysis buffer to extract DNA for microsatellite analysis (Otaño-Rivera et al., 2016). Used a chelex solution to extract DNA to genotype mouse models. Both proteinase K and chelex solutions are commonly used to extract and purify DNA from a range of tissues.

Of note, while the nucleic DNA extracted from the *shafts* of hairs (as commonly used in forensic protocols) may be substantially degraded, DNA from hair follicle cells originate from the epidermal and dermal layer of the skin (Paus and Cotsarelis, 1999). A well plucked hair will recover the hair bulb and sometimes a cuff of cutaneous tissue around it. The bulb is composed of a mixture of keratinocytes, melanocytes, fibroblasts and stem cells.

In the course of our work, we have compared a range of extraction protocols, based on both proteinase K and Chelex, to optimize yield of DNA from hair follicles. Here, we describe our production protocol that provides reliable DNA sequencing from hair follicles with low levels of chimerism, leveraging the Maxwell RSC 48 machine to partially automate DNA extraction (Senst et al., 2022; Dash et al., 2023). It combines a proteinase K cell lysis solution and the Maxwell RSC’s silica-based paramagnetic particles to purify DNA obtained from marmoset hair follicles. This protocol is broadly applicable to projects that may be looking to use hair follicles from any species, to use other low input DNA sources, or to simplify and automate DNA extraction of many samples.

Along with this experimental protocol, we demonstrate two computational methods to process the resulting data. First, we describe a method, based on mathematical distributions of allelic fractions at heterozygous sites, to measure chimerism from whole genome sequencing of a tissue; this method requires knowledge of only the host’s DNA (not the twin’s). For the second approach, we estimated the fractional proportions of the host and twin’s DNA from whole genome sequencing of a tissue. This second method requires knowing *a priori* the DNA sequence of the host and twin.

## 2 Materials and equipment

Promega’s Maxwell RSC instrument

Vortex and centrifuge

Thermal mixer for 1.5 mL centrifuge tubes

Cell Lysis Solution (i.e., Qiagen Puregene Cell Lysis Solution 158116)

Proteinase K 20 mg/mL (i.e., Qiagen 1019499)

DTT 1M

RNAse A (i.e., Qiagen 19101).

Maxwell RSC Tissue Kit AS1610

## 3 Methods

### 3.1 Animal use

Marmoset experiments were approved by and in accordance with IACUC protocol number 051705020. Hair follicles collected from eight rhesus macaques were used to optimize the protocol, and for empirical comparison to DNA from blood extraction. Macaque experiments were conducted under IACUC protocol #IP00004139. As a non-invasive source of DNA, the use of hair follicles was considered an ethical advancement over traditional blood or skin punch biopsies historically used in marmoset genetics.

### 3.2 Extraction of DNA from hair follicles

To obtain the hair follicles, select three small areas on the marmoset which remain relatively free of dirt or bodily fluids, ideally where the hair shaft is thicker. Examples include between the shoulder blades, nape of the neck, or upper lateral thighs. Use scissors to trim hair in the area to approximately ¼ inch. Wipe off trimmed hair so the area is clean. Using a hemostat or tweezers, grasp a clump of 10 trimmed hairs and gently pull against the grain to remove hair with follicles intact. Place the clump, follicle end first, into a 1.5 mL tube. Continue plucking hairs until approximately 50 hair follicles are in the tube. Repeat this process until three tubes with 50 hair follicles in each tube have been obtained, for a target total of 150 hairs per animal. Place tubes in −80 °C freezer.

To extract DNA, add 250 μL cell lysis solution to each of the 3 tubes. Vortex and spin down so hairs are at the bottom of the tube, submerged. To each tube, add 35 µL of Proteinase K and 5.5 µL 1M DTT. Pipette mix and spin down again to ensure hairs remain submerged. Heat tubes at 56 °C at 1,000 rpm for 1 h, spinning tubes down once in the middle. Most of the marmoset hair should dissolve completely. Pool the lysates from the 3 tubes into a single tube, leaving any intact hairs behind. Add 4 µL of RNase A to the combined lysate. At this point, you should have 1 tube per sample that contains the lysate from 3 tubes of hair.

To elute DNA using the Maxwell RSC instrument, follow the kit’s instructions to load cartridges. Add 70 µL of elution buffer to the elution tube. Transfer lysate to the first well, and follow the instrument’s instructions to run the instrument. When the instrument has completed, remove the elution to a clean 1.5 mL tube, leaving any resin behind. Store in −20 °C freezer, and send for sequencing.

### 3.3 Generation and primary analyses of whole-genome sequencing data

After extraction, DNA mass was quantified using a nanodrop spectrophotometer. Sequencing libraries were constructed using the NEBNext Ultra II kit (New England Biolabs, Ipswich, MA) and sequenced on an Illumina Novaseq with 2 × 150 bp paired-end protocol.

Illumina paired-end reads were aligned to the cj1700_1.1 reference marmoset genome assembly using bwa (Li and Durbin, 2009). Duplicate reads were marked using Picard Markduplicates, and GATK (McKenna et al., 2010) was used for SNP discovery and genotyping. Only bi-allelic SNPs were used in the analysis and the following filters were used: QD < 4.0 | FS > 60.0 | MQ < 40.0 | MQRankSum < −12.5 | ReadPosRankSum < −8.0 | MAF<0.01 | QUAL<500. SNP calls from all chromosomes were combined into one VCF file and additional filtering was performed to discard heterozygous sites that exhibited extreme allelic imbalance, i.e., the fraction of non-reference allele (from all samples) is less than 0.2 or greater than 0.8. In other words, this is a site-level filter; individuals may still have extreme VAFs at specific sites as long as the population level balance is preserved at that site.

### 3.4 Contamination analysis

The National Center for Biotechnology Information (NCBI) Sequence Read Archive (SRA) performs automated quality control on submitted datasets, including an assessment of species origin using the Sequence Taxonomic Analysis Tool (STAT). STAT is a k-mer–based classifier that compares sequencing reads against a curated reference database (NCBI RefSeq) to assign taxonomy and generate a taxonomic profile of the dataset. These profiles are used to validate the declared species in the submission metadata, detect potential contamination, and improve indexing and searchability within the SRA. We downloaded the STAT reports for the sequencing data described in this study, and summarized the species detected in the FASTQ file for each animal.

### 3.5 Chimerism analysis

To evaluate the extent of chimerism detectable in each sequencing library, we used two computational approaches.

First, we simply visualize the fraction of non-reference alleles at heterozygous sites. To do this, we used the genotypes and allele depth (AD) annotation in the VCF file obtained from the GATK variant calling pipeline. Only heterozygous sites with at least 30 observations (reads) were used in the analysis.

Second, we used a computational method called Census-seq to quantify the twin contributions. Census-seq has been successfully used to estimate the DNA contribution of each individual from whole genome sequencing data of a cell village composed of dozens of human embryonic stem cells (Wells et al., 2023). It uses the individual’s natural genetic variation as a barcode and finds the donor-specific mixing coefficients that maximize the likelihood of the observed DNA sequence data. Using an iterative expectation-minimization algorithm, Census-seq can determine the optimal mixture after a few iterations. Since Census-seq was designed for cell villages composed of dozens of individuals, it can be readily used on marmoset tissues where the number of individuals in the pool are between two to three.

To calculate the twin contribution Census-seq was used to analyze each sample with the following input parameters: the BAM file (alignment of the reads to the reference genome), the VCF file that contains genotype calls from the GATK variant calling pipeline and the list of the host and the twins ID in the VCF file. Prior to analysis, we filter the input VCF to retain only biallelic sites, where one allele matches the reference genome. Census-seq is available as part of the Broad Drop-seq tools on github: https://github.com/broadinstitute/Drop-seq. The detailed computational protocol for running Census-seq is available here: https://github.com/broadinstitute/Drop-seq/blob/master/doc/Census-seq_Computational_Protcools.pdf.

## 4 Results

### 4.1 Quality of DNA and sequencing data from hair follicle extraction

We calculated summary statistics of DNA yield and quality for 307 hair samples processed with the protocol (Supplementary Protocol; Supplementary Table S1). The total amount of DNA yield per hair sample was considerably lower than what is typically obtained from solid tissues or blood. The average mass of DNA was 0.49 µg (minimum 0.04ug, maximum 1.88ug). The median OD 260/280 without cleanup was 1.79 (minimum 0.99, maximum 4.89).

We selected 16 samples to characterize more carefully using Agilent Tapestation electrophoresis, loading 1.5µLof each sample. Of these samples, 9 yielded sufficient DNA for the Tapestation automated analysis to quantify (Supplementary Figures S1, S2). For these 9, the average DIN was 6.7 (range 6.3–7.2) and the modal fragment size ranged from 7.8 kb to 17.9 kb. This indicates that typical follicle DNA obtained by this protocol may be“intermediate integrity”: not as high molecular weight as fresh blood or cell line DNA, but better than degraded material that would necessitate a specialized single-strand or ancient DNA workflow.

Approximately 5%–10% of samples failed to yield detectable DNA with this protocol. This was attributed to failure of the hair sampling to obtain follicular cells and natural biological variation in DNA content due to the life cycle of hair, for instance seasonal shedding. Additionally, while optimizing this protocol, we used rhesus macaque hair samples from animals undergoing necropsy for other projects, commonly extracting 2.5–3.9 µg of DNA from 150 hair follicles (Supplementary Table S2).

**FIGURE 2:**
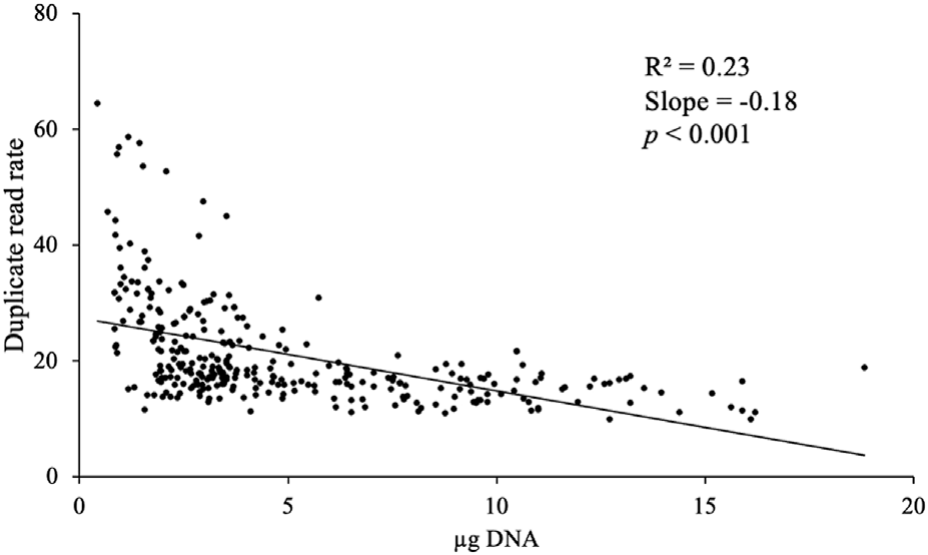
Duplicate read rate compared to µg of DNA extracted from 307 marmoset hair follicle samples using the protocol.

We found that the total mass of DNA available for each sample was predictive of the quality of the resulting sequencing libraries (Figure 2). The duplicate read rate, which is the rate at which the same DNA molecule is sequenced multiple times, increased with decreasing DNA mass. Libraries generated from more than 0.15 µg of DNA had an average duplication rate of 0.19 (range of 0.09–0.53, standard deviation of 0.07). In contrast, libraries generated from less than 0.15 µg of DNA had a higher average duplication rate of 0.37, with a much wider range (0.15–0.82, standard deviation 0.15).

As a reference, we compared duplicate read rates to that observed from high coverage whole genome sequencing of blood-derived DNA from rhesus macaques generated by our group, which have an average duplicate read rate of 0.257 (standard deviation of 0.108). Only nine marmoset hair samples fell outside 2 standard deviations of this mean, and eight of those samples were below 150 ng DNA, suggesting that a cut off of 150 ng DNA may be a reasonable minimum for decent library generation.

Finally, we looked at mapped read depth in 10 kb bins across the genome from hair follicles (n = 3), compared to skin samples isolated from marmosets (n = 3) and processed with the same protocol. Interestingly, while the correlation in read depth among all samples is high (0.90 averages among all samples), the correlation between samples from the same source is higher (average R for hair samples, 0.962, R for skin samples 0.957), indicating that there is indeed an effect of source on the pattern of mapped read coverage. When we examine the location of the largest discrepancies between hair and skin, we see that they are consistently mapping to the mitochondria, or apparent nuclear mitochondrial insertions (which are currently not well annotated in CalJac4 assembly). When we remove these sites from the analysis skin and hair look more similar (average R = 0.94 for all samples). Thus, a large part of the discrepancy can be attributed to increased MT DNA copy number in the skin compared to the hair follicle cells. This is consistent with a recent report that mtDNA quantity decreases spatially along the length of human hair shafts, while nuclear DNA is more stable (Brandhagen et al., 2018).

**FIGURE 3:**
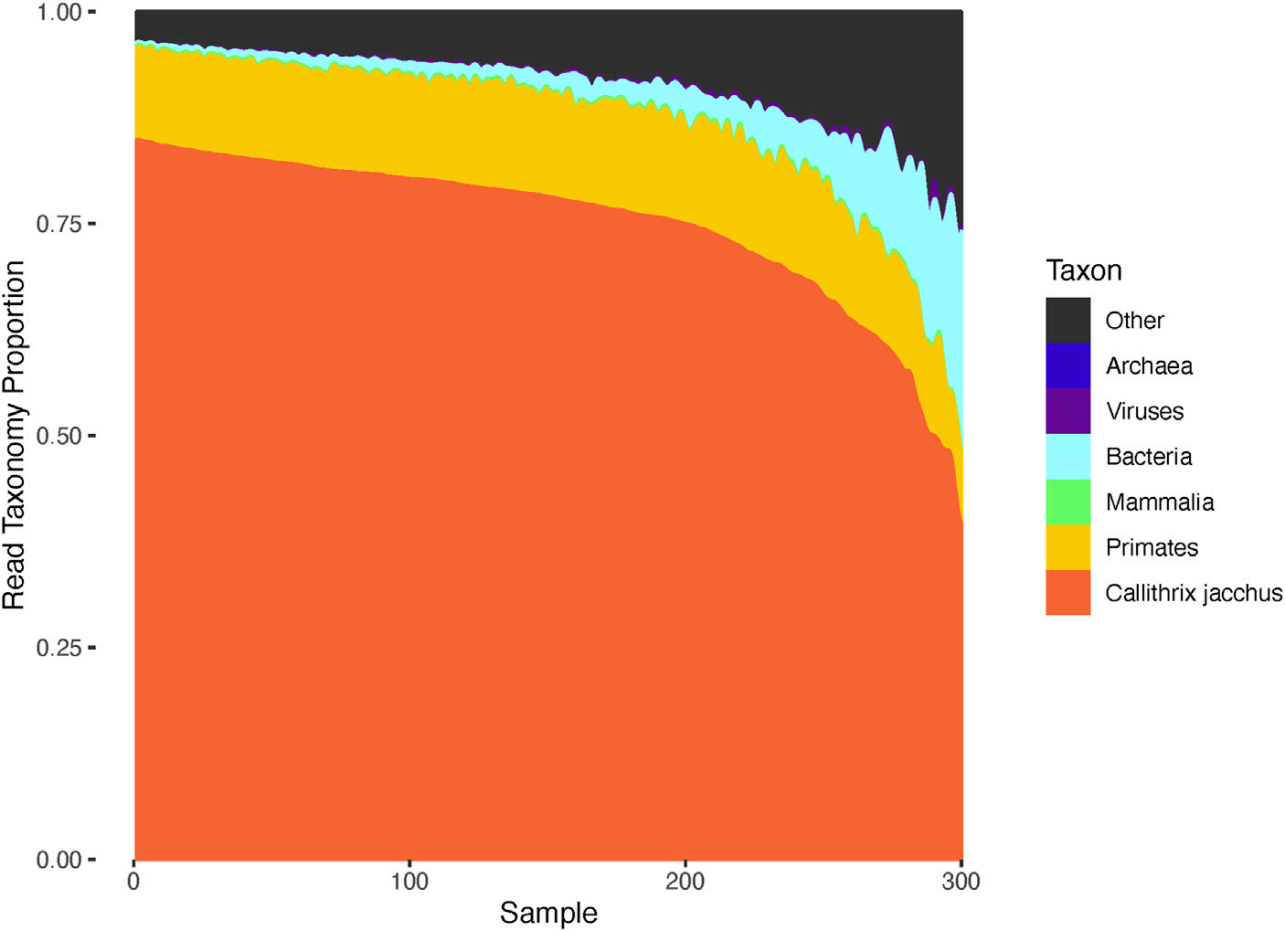
Species of origin estimated from each marmoset sequencing library. Analysis of taxonomy for the reads from each sequencing library identified a variable level of non-marmoset sequencing reads coming from microorganisms. The composition of each library is represented as a stacked barplot, with the bars (columns) representing distinct animals. On average 3.86% of reads per sample map to bacteria, with the maximum being 37%. Some reads are not confidently assigned to marmoset but can be assigned to close taxonomic levels such as “primate” or “mammals”. While most of these are probably marmoset, we could not exclude the possibility of human or other mammalian contamination. Likewise 2%–10% of reads fall in the “other” category which include low complexity and/or low quality sequence that is challenging to assign.

Using the automated results of the NIH SRA QC pipeline, we analyzed the species of origin of reads from each sequencing library (Figure 3). As expected, that majority of sequence data from each library was confidently assigned to *Callithrix jacchus*. Interestingly, a non-trivial fraction of reads came from microbes, especially bacteria. The average proportion of bacterial reads was 3.5% (standard deviation 4.8%). A small number of samples contributed the most contamination (23 samples had over 10% bacterial reads). Interestingly, *Corynebacterium stationis*, *Prevotella copri*, and *Bifidobacterium aesculapii* were the most abundant in terms of total reads identified, which accounted for 0.168, 0.126% and 0.66% of the total identified reads and observed in 52%, 100% and 100% of the samples sequenced, respectively. The bacteria phage Myoviridae sp. ctu2j3 was also notable with 0.20% of reads accounted for and found in 93.3% of the samples.

Notable, these three bacterial species are likely to be present on the skin and hair of the marmosets. Corynebacteria are classic skin/hair commensals across primates. Different *Corynebacterium* spp. dominate healthy scalp in humans, and primate skin/hair often shows Corynebacterium among top taxa (Kitrinos et al., 2022; Paul et al., 2025). *Prevotella copri* is best known as a prevalent gut bacterium (especially with plant-rich diets), not a skin organism. Finding it on hair most likely reflects transfer from feces or the environment (e.g., grooming, handling, cage surfaces) rather than a true hair/follicle resident. Notably, nonhuman primate hair microbiomes tend to be enriched for gut- and environmental taxa compared with human hair (Kitrinos et al., 2022). *Bifidobacterium aesculapii* species was first isolated from common marmoset feces. Its presence strongly suggests fecal transfer (Toh et al., 2015).

### 4.2 Empirical assessment of chimerism from hair follicles

**FIGURE 4:**
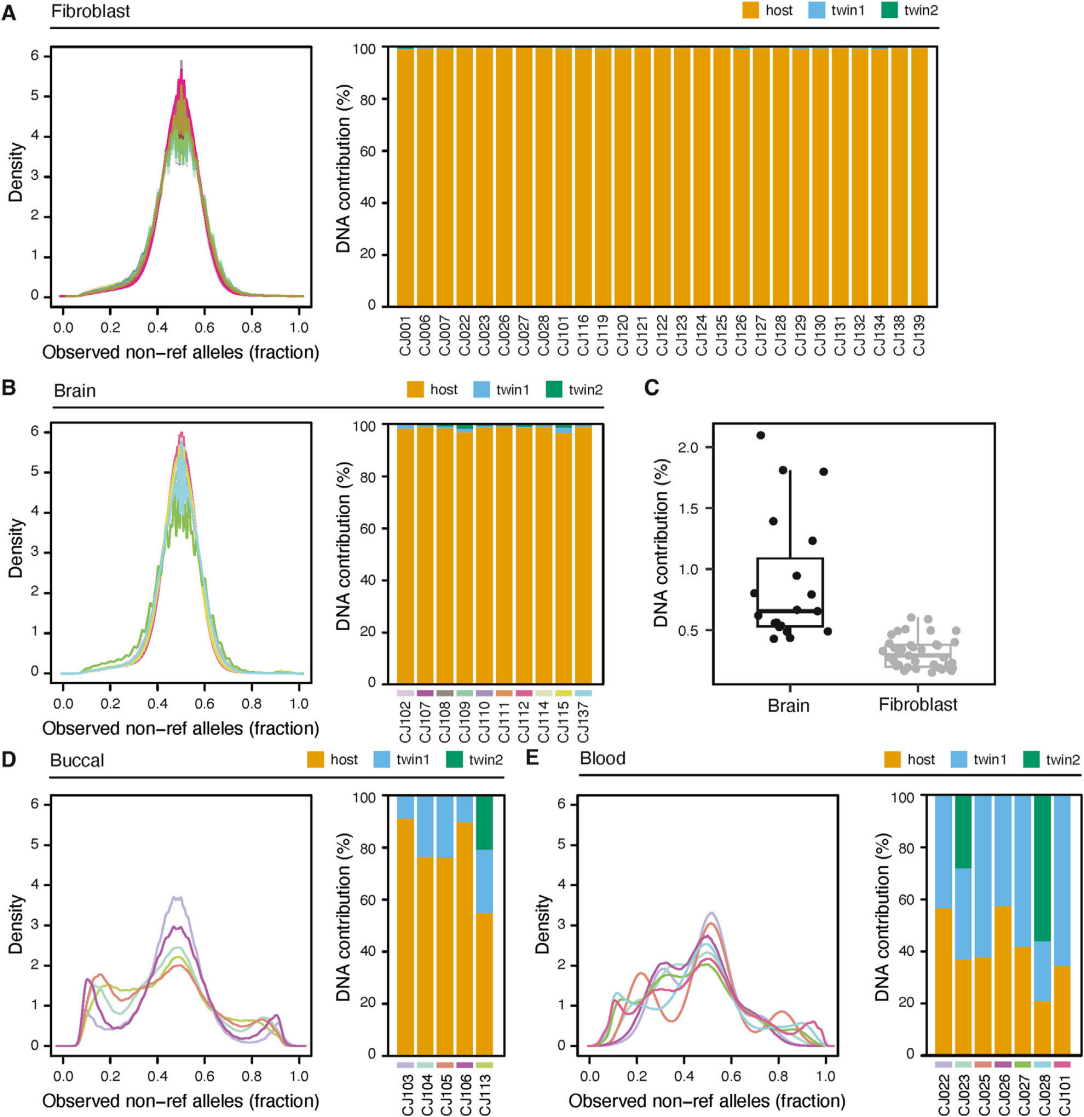
Assessment of chimerism in marmoset tissues. **(A)** left: Distribution of the fraction of non-reference alleles at each heterozygous site of an animal. Allelic fractions are from non-chimeric whole-genome sequencing of fibroblasts from 66 animals. Right: Census-seq estimates of chimerism fraction for a subset of animals whose twins were also sequenced. The estimated percentage of DNA coming from the host genome (“host”) is shown as a stacked barplot, along with the percentage coming from gestational siblings of the host (“twin 1”, and, in the case of triplets, “twin 2”). **(B)** As in **(A)**, but using perfused brain samples from 10 animals. Data from each animal is color coded to match between left and right panels. **(C)** Comparison of twin contributions between brain samples and fibroblast samples. **(D)** and **(E)** as in **(A)** but using buccal cells and blood, respectively.

We use two ways to assess chimerism - visual inspection of non-reference allele counts, and statistical estimation of twin chimerism using the Census-seq method. Census-seq requires genome sequencing data from both the host (the individual that is the subject of the analysis) and any gestational sibling(s). To provide context for the hair data, we used these methods to analyze published sequencing data generated from a variety of tissues sampled from 94 marmosets (del Rosario et al., 2024). Tissues included blood, skin, buccal cells, cultured fibroblasts and brain. At sites of known heterozygosity, we observed a unimodal non-reference allele fraction centered on 0.5 for cultured fibroblasts and brain, two tissues that have small or no contribution from hematopoietic lineages (Figures 4A,B). On the other hand, blood and buccal cells clearly show secondary peaks corresponding to twin DNA (Figures 4C–E). Census-seq estimates confirm these visual assessments (Figure 4): the estimated twin contribution to cultured fibroblasts (median 0.3%) and brain (median 0.7%) was significantly lower than to buccal cells (range 8.6%–45%) and blood (range 42%–80%).

**FIGURE 5:**
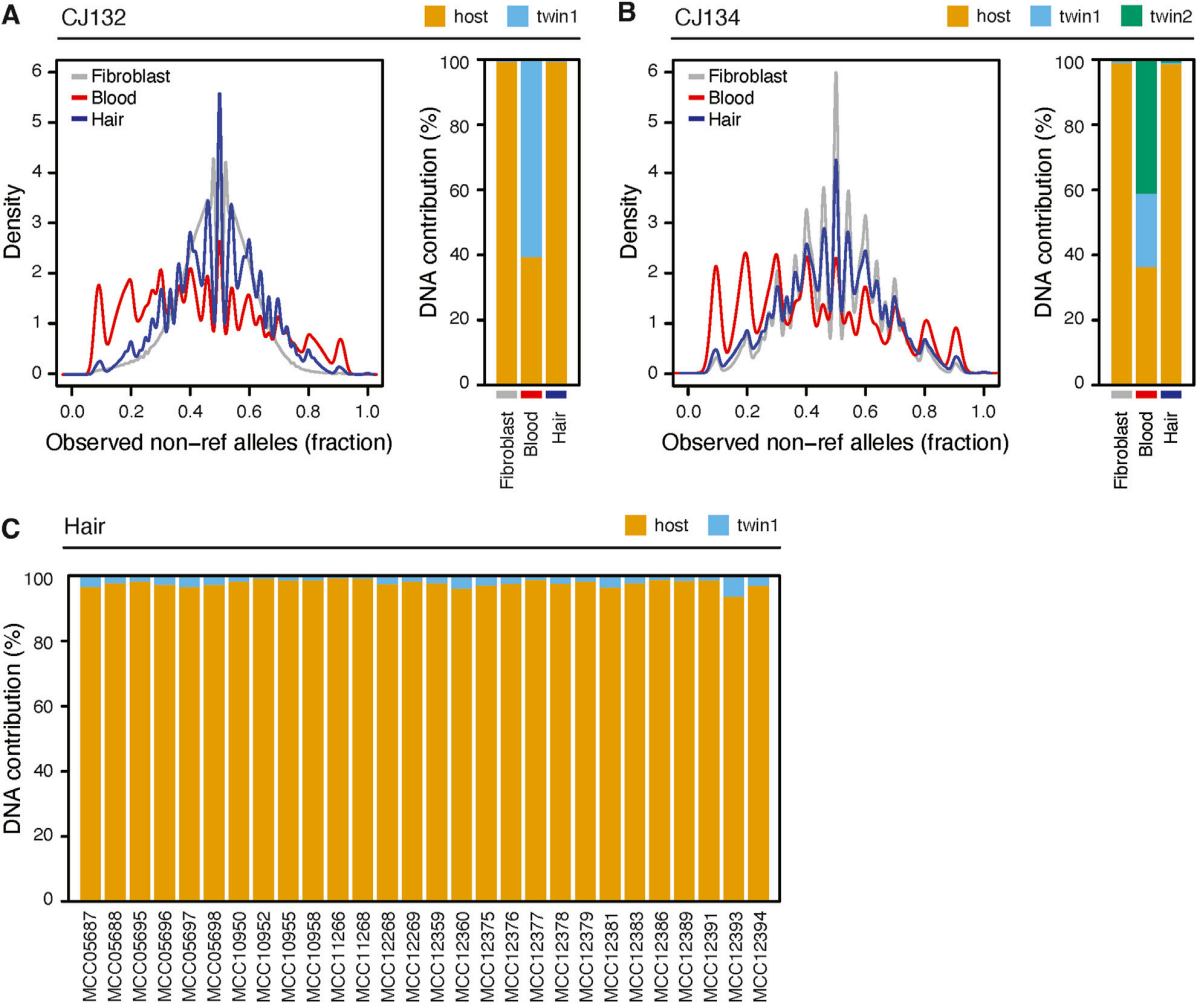
Hair sampling reduces chimerism compared to blood. **(A)** left: non-ref allele read fraction for libraries derived from hair, blood and cultured fibroblasts from animal CJ312. Right: census-seq estimates of chimerism fraction from the same samples. **(B)** As in **(A)** but for animal CJ134. Blood was sequenced at shallow coverage (7-11X). **(C)** Census-seq analysis showing the contribution of DNA from 28 sequenced individuals (“host”) and their gestational siblings.

Next we analyzed DNA prepared with the hair follicle protocol from two animals, CJ132 and CJ134, comparing it to matched blood and fibroblasts, and found that hair DNA is largely free from chimerism (Figures 5A,B). These samples were sequenced at a lower coverage, 7-11X, and thus the alternate read distributions appear more granular. Even at shallow sequencing, the difference in fibroblast and blood profiles are visually evident, and more notably, there was almost no difference between fibroblast and hair. Using Census-seq, we estimated the chimerism fraction of CJ132s hair to be 0.52%, while the contribution of each of CJ134s twin to hair follicle DNA were 1.04% and 1.81% (Figures 5A,B). Finally, we analyzed the WGS sequencing data from 28 sibling pairs (Figure 5). These animals were sequenced to approximately 30X coverage. Chimerism analysis indicated that birth siblings contributed an average of 2.3% of the DNA sequenced from an individual, confirming that the hair follicle DNA extraction protocol can be broadly effective in limiting chimerism. No birth sibling contributed greater than 6.5% of DNA.

## 5 Discussion

Using hair follicles is a non-invasive way to extract DNA from marmosets with minimal chimerism. We were able to routinely extract DNA from 150 hair follicles suitable for high-depth whole genome sequencing. The DNA extracted from hair follicles had an average rate of chimerism of 2.3%, indicating that hair follicles contain very low levels of chimerism and are an excellent tissue to use for sequencing to avoid confounding sibling DNA. Additionally, obtaining hair follicles is not invasive, can be performed at yearly check-ups, and can be easily sent through the mail for collaborative research.

To maximize the likelihood of success of DNA extraction, several steps are critical. The most important is that a hair follicle is present at the end of the hair shaft. Hairs should be plucked carefully to ensure the shaft remains intact and the entire follicle is removed. We found higher success rates when the hairs were trimmed to a quarter of an inch on the animal prior to plucking, both to ensure better follicle retrieval (less shaft breakage) and to reduce the amount of debris that needs to be purified. Hair follicles, like any DNA source, should be kept on ice or frozen to preserve DNA integrity.

This method uses approximately 150 hair follicles to maximize chances of a successful DNA extraction while minimizing the invasiveness to the animal. To preserve DNA integrity and reduce time between the freezer and DNA extraction, there was no direct quantification in hair follicles received from each center. Additionally, veterinarians at each center had discretion in where to sample and how much hair to send, so there may have been variation from center to center in the total amount of hair follicles actually used in sampling. Additionally, while we found that 150 hairs was enough material to have a high likelihood of generating a library, adequate DNA has been extracted from as low as 30–50 hair follicles using this method, indicating that there is flexibility in the number of hair follicles used. Future studies could include more precise quantification of input tissue, and analysis of location on the animal to better understand the nuances of DNA extraction and subsequent quality of sequenced DNA.

Ancient DNA analysis is another research area that requires isolation of nucleic acid, often highly degraded, from a scarce amount of input material, including bone, teeth and hair shafts (Rohland et al., 2010; Kapp et al., 2021). In the ancient DNA field, it was observed that single vs. double strand DNA extracted from samples vary in generating quality libraries for sequencing (Gansauge and Meyer, 2013; Gansauge et al., 2017). We believe that, when isolating DNA from well-plucked hair follicles that have been processed quickly and stored well, we should be isolating primarily dsDNA. Additionally, silica based extraction (used here in the RSC cartridges) has been noted to extract predominantly dsDNA (as compared to chelex, which favors ssDNA). While we did not specifically look into the proportion of strandedness, based on the literature this protocol likely favors dsDNA (Stoljarova-Bibb et al., 2025).

In addition to methods for DNA extraction, the ancient DNA field has also innovated news methods for sequencing library preparation from limited and damaged DNA (Rohland et al., 2010; Kapp et al., 2021). It should be noted here that in this study we used a general library preparation kit from a commercial vendor; while the vendor advises that the kit is suitable for small amount of DNA (as little as 500 pg) from challenging samples (e.g., FFPE samples), it is quite possible that we could obtain better results (e.g., lower duplicate read rate, more uniform coverage) if we used a more specialized library prep method such as those intended for ancient DNA.

Importantly, we demonstrated that the quality of the resulting sequencing library can be somewhat predicted from the starting DNA mass. As the total starting mass drops, there is an increasing chance of a low complexity library and thus low efficiency in genome sampling. Another potential concern we have seen with the lowest input DNA samples is the presence of competing DNA contamination from microbes on the hair itself. As described above, microbial DNA contamination can be detected and should be controlled at the analysis stage of a genome-sequencing workflow.

We described the use of a published analysis pipeline, Census-seq, for estimating chimerism fractions from marmoset DNA. Using genetic variation to analyze mixtures of DNA is a well established problem, with specific applications in detecting contamination in high-throughput sequencing facilities(Fiévet et al., 2019; Balan et al., 2023), and in characterizing the composition of DNA samples in forensic investigation(Gill et al., 2021). We found that most published methods are human-centric, assuming population variation databases, and in the case of forensics often rely on STR data (instead of next-gen sequencing). The Census-seq approach is applicable to 2 or more individuals of any diploid species, if appropriate short-read sequencing data is available for each individual and has been aligned to a reference genome.

The partial automation using the Maxwell RSC instrument increases efficiency and accuracy. While the fully manual method requires approximately 6 h of hands-on work, including 2.5 h of total incubation time, the partially automated method is a total of ∼3 h, with 1 h of hands-on time, and 2 h of incubation. Additionally, the partial automation allows for more samples to be processed at once—the Maxwell RSC 48 can process up to 48 samples at one time (though we recommend halving that to prevent potential contamination by splashing). Similarly, because of the automation, the likelihood of human error and batch effects are reduced. The partial automation afforded by the Maxwell RSC is a valuable tool that decreases time spent in the lab and reduces variability between extractions.

In the development of this protocol, other sequencing methods were evaluated, including Chelex protocols, manual extraction using glycogen, and Qiagen tissue extraction kits (see Supplementary Material). Generating quality DNA from the small amount of tissue from hair follicles was the biggest challenge for most of these methods, however other evaluated factors include reproducibility, time, and contamination risks.

Because the Maxwell RSC instrument allowed for partial automation, time spent per sample and reproducibility were improved. Additionally, we also found the Maxwell RSC instrument to produce DNA that was comparable in quantity and purity to manual methods. A completely manual extraction, using proteinase K and glycogen, was the most effective manual solution, with high quality DNA. The main drawback was the long duration (up to 8 h per set of samples) which was not ideal for a large project. The Chelex method resulted in lower DNA yields and more contaminated DNA, likely due to Chelex retaining some DNA even when eluting. Qiagen tissue kits resulted in purer DNA, but also had lower yields, even with minimal washing of spin columns. Detailed protocols for these methods are in the Supplementary Material.

While this protocol was designed to solve the problem of chimerism in marmoset blood, it is broadly applicable to other species and would be useful in other scenarios where obtaining a blood or tissue sample may not be possible. We demonstrated success using hair follicles from macaques to generate enough DNA for whole genome sequencing library preparation. This protocol was developed to generate DNA for WGS, but could be adapted for additional sequencing applications, such as targeted sequencing or methylation studies. The primary concern for the short term is the required input for downstream methods, such as the quantity of DNA, the length of the DNA fragments, and the number of cells. We see the method as being immediately useful for targeted-short read sequencing and methylation sequencing. As we demonstrated, the fragment sizes obtainable from this protocol (modal fragment size of 8–18 kb) would make the resulting DNA useful for long-read protocols, although not as good as high-molecular weight DNA from cell cultures.

In conclusion, DNA extraction from 150 hair follicles using the Maxwell RSC 48 generates DNA suitable for WGS with minimal chimerism in marmosets. This method offers partial automation, reducing hands-on lab time and batch variability.

## Data Availability

The raw whole genome sequencing data described in this project are available from the NIH Sequence Read Archive, under accession number bioproject PRJNA1068102.
