# Supplementary material for: A partially automated method for DNA extraction from marmoset hair follicles to avoid blood chimerism
Source: Front Genet. 2025 Sep 26;16:1608504. doi: 10.3389/fgene.2025.1608504 (PMC12510806; doi:10.3389/fgene.2025.1608504)
Supplement: Supplementary file 2 [file DataSheet1.docx]

***Supplementary Material***

# Supplementary Data

# Supplementary Method 1: Manual extraction

**Materials:**

- Qiagen Cell Lysis Solution – 158906
- Qiagen Rnase A – 158922
- Qiagen Protein Precipitation Solution – 158910
- Proteinase K – NEB P8107S (Any brand should work as long as input is matched) - 800units/ml
- Glycogen – R0561
- 100% Isopropanol
- 100% Ethanol for Molecular biology
- Distilled water, TE buffer, or any preferred DNA buffer

**Method:**

1. To plucked hair add 400 uL Qiagen Cell Lysis Solution. Cap and vortex for a second or two. Spin down.
2. Add 35 uL Proteinase K. Shake well by inverting.
3. Incubate at 56^o^C at 1000rmp for 30 min. Invert a few times and incubate for 30 more minutes; 1 hour total incubation.
4. Change incubator to 37C
5. Spin down well, remove as much liquid as possible with a pipette and place in a new tube, leaving the hair or other chunks behind to be discarded. The DNA from the follicle should be in the solution.
6. Add 4 uL of (1.5ul Qiagen Rnase A), cap and invert about 20 times to mix.
7. Incubate at 37^o^C for 30 minutes.
8. **While waiting** Place Qiagen Protein Precipitation Solution on ice. Place 100% Isopropanol on ice. Chill a microcentrifuge to 4 degrees. Get out Glycogen and thaw on ice.
9. When incubation is done, spin down and place tubes on ice for 5 minutes.
10. Add 300 uL Protein Precipitation Solution to each tube.
11. Pulse vortex for 25 seconds. Sample should turn milky white.
12. Spin at 13,200 rpm for 10 minutes at 4^o^C to pellet precipitated protein.
13. **While waiting** Prep new 1.5 mL tubes with labels for every sample
14. Prep mastermix of 400ul cold 100% Isopropanol + 0.25ul glycoblue for each sample (keep on ice or return to freezer while waiting)
15. Add 400ul to each newly labeled tube (and keep cold)
16. When the centrifuge is done, move quickly to pour off only the supernatant from the spun down tubes into the corresponding tubes containing Isopropanol/Glycogen. Some people use a pipette for this, but just doing one single pour motion into the alcohol tube and holding the pour off tube upside down over it for a few seconds works best. You don’t need to pour off every last drop of supernatant because you want to avoid transferring the white protein pellet into the alcohol containing tube. There is always some remaining supernatant around the protein pellet and there is no need to recover this. Discard the tubes that now just have a protein pellet. **Be careful NOT to pour the Isopropanol containing tubes into the pelleted protein tubes.**
17. Shake the supernatant mixed with the Isopropanol tube well. When large amounts of DNA is present you can see it precipitate here.
18. Freeze overnight
19. Spin at 13,200 rpm for 10 min at 4 degrees C to pellet the DNA.
20. When done spinning, carefully pour off all the liquid in one smooth motion into your waste container. Don’t worry about the pellet it will stay even if you can’t see it. While pouring the samples one by one take the poured off tube and, keeping it upside down the whole time, rest it upside down against a tube rack on some napkins, which allows some extra isopropanol to drain out while you pour off the others.
21. When all samples are poured off, place them back upright in your tube holder, and add 300 uL of freshly prepared 70% Ethanol to wash the pellet. You usually have to smack the tube pretty hard against the lab bench a few times, which dislodges the pellet (that you can sometimes see and sometimes can’t).
22. Spin at 13,200 rpm for 5 minutes at 4^o^C to re-pellet the DNA.
23. Turn on incubator to 37C
24. When the spin is done, again in one smooth motion pour off the ethanol, and keep the tubes upside down on paper towels to let extra ethanol drain off as you go.
25. One tube at a time, while still keeping the tube upside down, take either a lab swab or twisted up kimwipe and wipe out the excess Ethanol from around the sides of the tube. Be careful to NOT wipe near the very tip of the tube where the pellet is going to be. Try to get as much ethanol out of the tube so that you don’t have to air dry for a long time, but you can just let them air dry if you have to.
26. Flip the tube(s) upright in a tube rack and let dry (can incubate at 37C) until no Ethanol is visible at all. Ensure no ethanol remains!
27. Rehydrate the gDNA with about *20 uL of Qiagen Elution Buffer (EB). Flick the tube to mix and spin down. *Large pellets may require much more rehydrating buffer, or desiring very concentrated DNA would use less. You can alter this amount to what suits your needs when you have an idea of your DNA returns after a few extractions. It is better to add less water at first because you can always add more if you need the final DNA to be less concentrated.
28. Allow the gDNA to rehydrate at 55^o^C for 15-60 minutes depending on the pellet size and how much water you added to rehydrate. Leave lids open so any remaining ethanol can evaporate.
29. No pellet should be visible when the DNA is fully rehydrated.
30. Proceed QC

#

# Supplementary Method 2: CHELEX extraction

Adapted from Singh et al 2018<https://link.springer.com/article/10.1186/s12575-018-0077-6>

**Materials**

Chelex 100 Resin (BioRad, Cat no #142–1253)

Ammonium acetate *it comes dry

Sodium acetate

Isopropanol

Ethanol

1X TE buffer

Make buffers

10% Chelex suspension (pH 8.0, to be prepared in 1X TE buffer)

- 10% (wt/vol) Chelex 100: Add 1 g Chelex 100 resin to TE Buffer and bring up to a final volume of 10 ml.

7.5 M Ammonium acetate

- Dissolve 5.7g of ammonium acetate in water to a final volume of 10ml. Sterilize by filtration (0.2µm filter). The final pH will be 5.5.

3 M Sodium acetate

- 2.5g + fill to 10ml H2O for 3M

(Equation for 7.5M --> 7.5mol/L * (molar mass)g/1mol * (amount to make ie 0.01)L = grams

**Before starting**

- Heat water bath to 100°C
- Chill 100%, 75% ethanol in -20C
- 100% ice cold isopropanol
- Make buffers

**Protocol *Alt = mouse hair follicle protocol**

1. Thoroughly vortex chelex stock solution, pipette out immediately
2. Add 200ul 5 and 10% chelex solution to empty 2ml tubes (Alt: 10%)
3. Boil tubes (no tissue yet) for 10 minutes at 100C in water bath. Tube lids may pop open, ensure no water gets in.
4. While chelex is boiling, wash tissue with 2 ml of 1X PBS (X2) – centrifuge after second wash to completely remove PBS
5. Add hairs to boiling Chelex solution. Boil for 15 minutes, vortexing in the middle. Tube lids may pop open, ensure no water gets in. (Alt: 20-40 min)
6. Briefly centrifuge tubes to ensure all liquid is at the bottom
7. Pipette out all liquid into new tube (this step removes hair)
8. Centrifuge tubes at 12000g for 1.5 minutes
9. Pipette out supernatant (avoid chelex resin)
10. Add 4ul RNAse A (100mg/ml RNAse)
11. Measure 1ul on Nanodrop for quality control

Protein extraction

1. Measure supernatant (X)
2. Add 1/2X of 7.5M stock of ammonium acetate. A thick yellow precipitate may form.
   1. 7.5 M stock solution of ammonium acetate to the supernatant (so working conc. Ammonium acetate in solution is 2.5M).
   2. 7.5M * v = 2.5 (X + v)
3. Rest on ice for 5 minutes
4. Vortex for 5s
5. Centrifuge at 12000 rpm for 10 minutes at RT
6. Collect clear supernatant (containing genomic DNA)

DNA precipitation

1. Measure supernatant (X)
2. Add 1/9 X of 3M sodium acetate stock to supernatant so working concentration of sodium was 0.3M in solution -- beware that the concentration of ethanol in the next step will change depending on the volume we have, so take that into consideration if we decide to use 600uL
3. Add 200ul of ice cold 100% ethanol
4. Add 0.01X Glycoblue (15 mg/mL) to a final concentration of 150 µg/mL
5. Vortex
6. Store overnight in -20
7. Centrifuge at 15000g for 1h at 4C
8. Discard supernatant, KEEP PELLET
9. Add 1ml ice cold 75% EtOH to tube without disturbing pellet
10. Centrifuge at 15000g for 10 min
11. Repeat 8-10
12. Add 1ml 100% ice cold isopropanol
13. Remove (immediately) as much as possible without disturbing pellet
14. Air dry pellet in laminar flow hood for 7-10 min
15. Add 20ul buffer EB, incubate at 55C for 10 min to facilitate solubilisation

Supplementary Material should be uploaded separately on submission. Please include any supplementary data, figures and/or tables.

Supplementary material is not typeset so please ensure that all information is clearly presented, the appropriate caption is included in the file and not in the manuscript, and that the style conforms to the rest of the article.

# Supplementary Figures and Tables

For more information on Supplementary Material and for details on the different file types accepted, please see [here](https://www.frontiersin.org/guidelines/author-guidelines#supplementary-material).

## Supplementary Figures


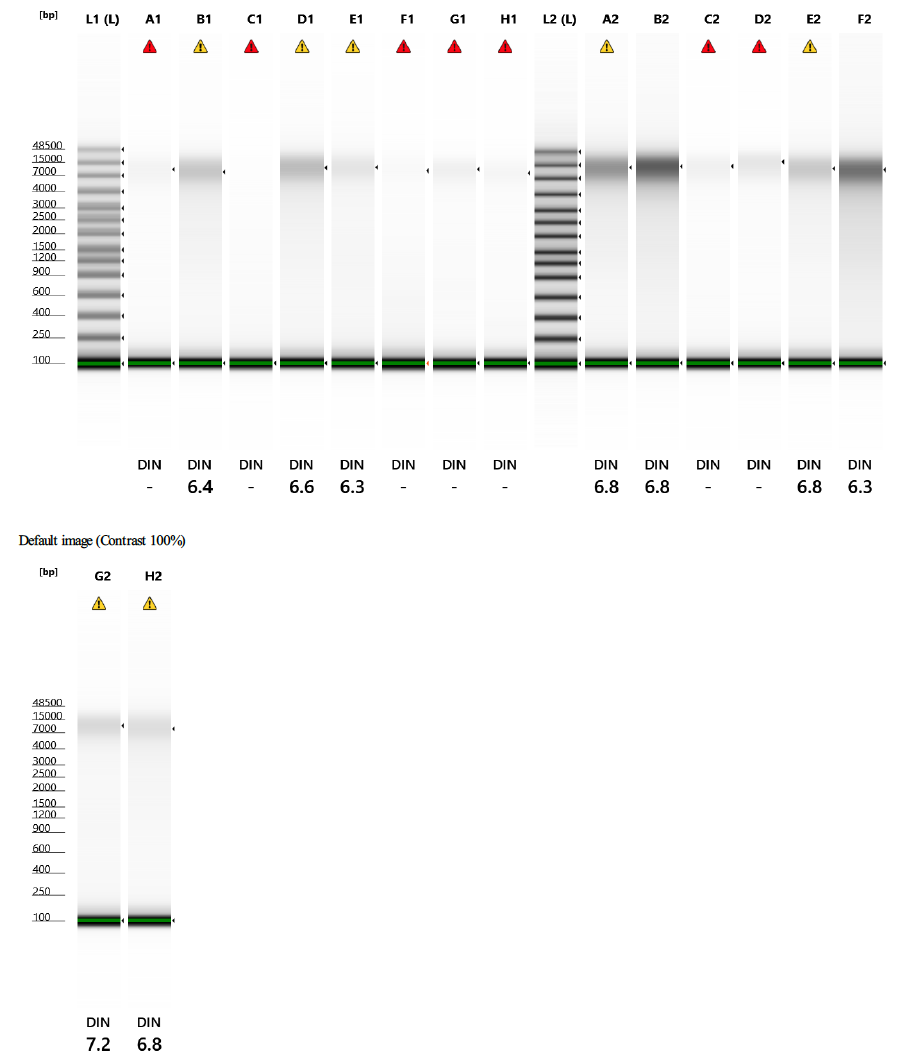


**Supplementary Figure 1.** Agilent Tapestation output for 16 follicle samples run with D1000 ScreenTape. 1.5uL of sample was loaded from each sample. L1=ladder, L2=ladder, all other lanes are sample. The yellow triangles indicate samples outside the recommended range of DNA concentration, and red triangles are sample with DNA concentration that are too low to be analyzed. Computed DNA integrity scores (DIN) are shown at the bottom of each lane.


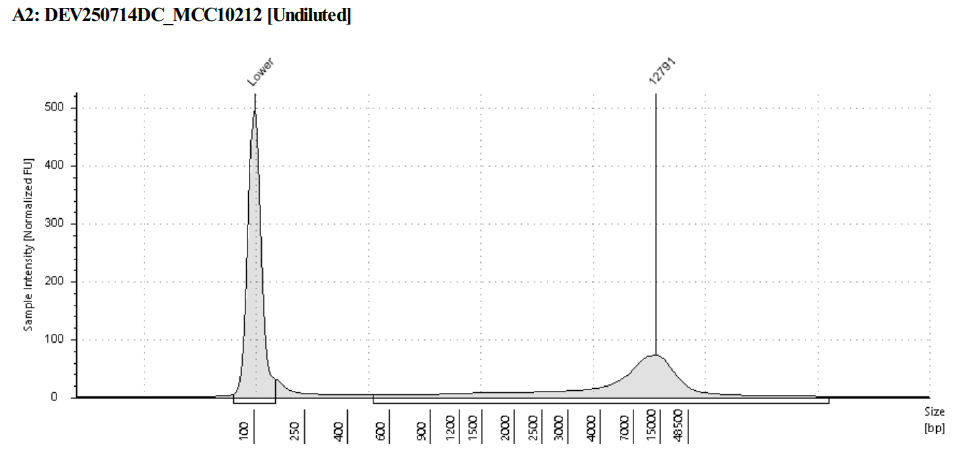


**Supplementary Figure 2.** Representative peak trace for one sample (lane A2). The median peak size is 12,791bp, with 95% of the integrated sample area falling between fragments of 507bp to over 60kb. The DIN value for this sample was 8.44 and estimated concentration 8.44 ng/uL.
